# Analysis of the Future Evolution of Biocapacity and Landscape Characteristics in the Agro-Pastoral Zone of Northern China

**DOI:** 10.3390/ijerph192316104

**Published:** 2022-12-01

**Authors:** Xiaoyu Niu, Yunfeng Hu, Lin Zhen, Yiming Wang, Huimin Yan

**Affiliations:** 1 State Key Laboratory of Resources and Environmental Information System, Institute of Geographic Sciences and Natural Resources Research, Chinese Academy of Sciences, Beijing 100101, China; 2 School of Geosciences, Yangtze University, Wuhan 430100, China; 3 College of Resources and Environment, University of Chinese Academy of Sciences, Beijing 100049, China

**Keywords:** land use/land cover, biocapacity, landscape index, model simulation, dynamic change, forecasting method

## Abstract

The Agro-Pastoral Zone of Northern China (AZNC) is an ecologically fragile zone. It is a challenge to create scientifically sound plans for environmental conservation and agro-pastoral development due to the lack of future evolution prediction, and analysis of biocapacity (BC) and landscape characteristics. Using the Globeland30 dataset from 2000 to 2020, this study simulated 2030 land use/land cover (LULC) scenarios, and analyzed the future evolution of BC and landscape patterns. The results show that: (1) The Logistic and CA-Markov models can reasonably simulate the LULC changes in the research area, with ROC indices over 0.9 and Kappa approaching 0.805, after considering the driving factors such as physical geography, regional climate, and socio-economic development. (2) From 2000 to 2030, the spatial distribution pattern of LULC does not change significantly, and cultivated land, grassland, and forest are still the dominant land types in the research area. The regional BC exhibits an increasing trend (+4.55 × 10^6^ gha/a), and the spatial distribution pattern of BC is similar to that of LULC. (3) Changes in land miniaturization, landscape fragmentation, and decreased aggregation can be seen in the entire AZNC and specific land categories, including cultivated land, grassland, and forest. The study provides suggestions for formulating the AZNC’s future ecological protection and agro-pastoral development strategies, and guidance for the LULC simulation in other agro-pastoral zones.

## 1. Introduction

The Agro-Pastoral Zone of Northern China (AZNC) is the region that sits between agriculture in the east and its livestock regions in the west. The study area is spatially interspersed with cultivated land, grassland, and forest in a nested distribution. The AZNC is a significant ecological barrier belt which is crucial in limiting the expansion of desert land, and reducing sand and dust storms. Large climatic fluctuations and human land exploitation will alter the status of land use/land cover (LULC), and the composition and spatial characteristics of the landscape, leading to changes in regional ecosystem structure and ecosystem services. Therefore, it is crucial to analyze and predict the change process of LULC, biocapacity (BC), and landscape feature in AZNC. This will help the study area to formulate scientific and reasonable land exploitation and ecological conservation strategy, and promote regional sustainable development in terms of ecological security, food security, and resident livelihood security.

In terms of LULC change simulation, researchers have created many models for LULC change prediction, including the Cellular Automaton (CA) [1], Support Vector Machine (SVM) [2], CLUE-S model [3,4], CA-Markov model [5], and Artificial Neural Network (ANN) [6]. Although the CA-Markov model has a robust time–space simulation computation capability, driving forces are not considered [7,8]. The simulation results of the CLUE-S model are easily influenced by subjective factors and are best suited for small-area and fine-scale simulations [9]. The nonlinear change process can be simulated using ANN. However, its derivation and simulation techniques are opaque [10]. The logistic regression model can quantitatively describe the correlation between LULC changes and its driving factors, and can predict the probability of occurrence of a specific type of land. However, the logistic regression model can only simulate quantitative changes rather than simulate spatial changes [11]. In order to run the simulation process faster, better understand the mechanism, and make the simulation results more reliable, researchers need to use multiple models simultaneously. For example, Wang et al. [12] used the Logistic-CA-Markov model to predict the future (2015–2042) variation trend of LULC in the Qilian mountain area. The results showed that, compared with the AHP and MCE models, the contribution (weight) of each driving factor to the change in LULC obtained through the logistic regression model was more objective. Gharaibeh et al. [13] combined ANN with CA-Markov to predict LULC change scenarios in Irbid, Jordan. The results showed that the Kappa index of the ANN and CA-Markov ensemble model is 90.04%, which is much better than the CA-Markov (86.29%) model.

Researchers have sequentially put forth ideas like the Ecological Footprint (EF), BC, and Human Appropriation of Net Primary Production (HANPP) in terms of BC accounting and evaluation [14,15,16,17,18]. From the viewpoint of urbanization, Rashid et al. [19] analyzed the EF of Bahria town and Gulraiz colony in Pakistan. The findings demonstrated that the study area’s EF was higher than the national standard value and that resource consumption was higher than the region’s carrying capacity. The Driving-Pressure-State-Impact-Response model and the Analytic Hierarchy Process were used to create a BC evaluation index system by Bao et al. [20]; it was found that the BC composite index of Xilingol in Inner Mongolia showed an upward trend between 2000 and 2015. HANPP was initially used to demonstrate the extent and effectiveness of land use [21]. HANPP is now used to quantify the loss of ecosystem service value (ESV) [22] and measure the potential for regional sustainable development [23]. Because of its distinct meaning, straightforward approach, and mutual reference across scales and countries, BC is the most extensively utilized indicator for BC assessment.

Changes to parameters like LULC, yield factor (YF), and equivalence factor (EQF) during the quantitative calculation of BC will affect the computation of regional BC. Wang et al. [24] used the CA-Markov model to simulate future LULC under several LULC change scenarios of northern Shaanxi in the Loess Plateau, as well as the spatio-temporal evolution of the local BC. Based on information on agricultural yields provided by the Food and Agriculture Organization of the United Nations, Wackernagel et al. [25] were the first to determine the EQF of various land types worldwide. The World Wildlife Fund (WWF) assessed the EF and BC at the global scale and some countries over time and provided pertinent calculation parameters in its “Living Planet Report”. Liu et al. [26,27] estimated YF and EQF for various land types in China and Chinese provinces based on the net primary productivity of vegetation and MODIS data. The BC indicator is a numerical value based on the kind of ecosystem and the characteristics of the area. However, BC does not consider spatial changes of the ecosystem, let alone those that may influence the ecosystem service’s capacity, vulnerability, and resilience. To more accurately assess the effects of ecosystem type composition and spatial structure on ecosystem service functions, it is required to further integrate the analysis of changes in ecosystem landscape characteristics while conducting the BC evaluation.

Landscape ecology studies the spatial structure, interactions, coordination mechanisms, and dynamic changes of an ecosystem or landscape. There are several GIS-based tools for calculating landscape pattern indices in recent years, including Fragstats [28], PolyFrag [29], and VecLI [30]. Dadashpoor et al. [31] analyzed the impact of LULC changes and urbanization on the local landscape pattern in the Tabriz metropolitan area based on remote sensing images from 1996 to 2016. The research results show that, from 1996 to 2016, most of the ecological land such as grassland in the study area was transformed into construction land and bare land, which led to the intensification of landscape fragmentation and the decline of landscape in the study area. Using remote sensing images, Zhao et al. [32] studied the impact of LULC and landscape pattern changes on local ESV in Guizhou Province. The results showed that the LULC and landscape pattern in Guizhou Province has changed significantly from 2000 to 2017; cultivated land and grassland decreased, water body and construction land increased, and forest land increased first and then decreased. The two major landscape types of cultivated land and grassland in the study area were gradually replaced by forest land and water bodies, which resulted in the ESV loss of up to 1.63 × 10^9^ RMB in the study area. In general, the majority of current studies use the landscape index to examine the dynamic change process of landscape pattern, its driving factors, and effects on ESV. In addition to these conventional studies, more researchers are focusing on the impacts of changing landscape characteristics on the functioning of ecosystem services. For instance, Hu et al. [32] examined the effects of spatial layout patterns of urban green spaces on urban heat islands at the Olympic Village in Beijing, China. Wilson et al. [33] examined the effects of landscape fragmentation on multi-scale biodiversity. The findings revealed a significant correlation between species richness at the patch scale and habitat occupancy in the landscape, and small islands could accumulate species faster at the landscape scale. In conclusion, it is crucial for regional BC and ESV studies to consider the overall changes that are primarily dependent on the kind of landscape and the qualitative changes brought by various aspects of the spatial layout of the landscape.

Since the late 1990s, the Chinese central government has implemented a series of ecological protection and restoration initiatives in the AZNC, including the Beijing–Tianjin Sandstorm Source Control Project, the Three Norths Shelter Forest Project (Phase IV), and the Conversion of Farmland to Forests and Grasses Project. The “China’s Major Function Oriented Zone,” published by the State Council of China in 2010, made it clear that the major functions of the AZNC are “wind and sand fixation” and “ecological barrier”. Large-scale ecological construction projects, particularly the Conversion of Farmland to Forests, basically ended after 2015. Governments have seriously taken regional food security concerns, and the alleviation of poverty of farmers and herders. The LULC evolution of the AZNC, its ecological advantages, and the possibility of ecosystem evolution have attracted significant attention from scientists worldwide [34,35]. Fu et al. [36] evaluated quantitatively the impact of the policy of converting farmland to forests on ESV in the Loess Plateau. The findings showed that, between 2000 and 2008, the farmed land transitioned to grassland and forests, which improved the area’s ability to conserve soil and water, and sequester carbon. However, the socio-economic incentives in the ecological restoration policy led to an increase in food production even if the amount of farmed land shrank. Hu et al. [37] analyzed the Loess Plateau’s crucial ecological service functions, and examined the effects of future climate change and human land development activities. The findings indicated that the rigorous ecological protection scenario would result in a decline in grain production but an increase in the capacity for grazing livestock. All the other LULC change scenarios would result in simultaneous reductions in food production and sustainable stocking capacity. Therefore, the synchronized economic and ecological development scenario is the most advantageous for the region’s long-term sustainability. On the one hand, this scenario ensures the fundamental stability of the local staple food production. On the other hand, despite a modest decrease in the sustainable stocking capacity, the significant gain in habitat quality can compensate for its negative impact. In conclusion, most research concentrates on the Loess Plateau, regardless of whether they are general LULC studies, or BC and ESV change studies based on the LULC change scenario. Less study has been done on the simulation of LULC and its potential effects on the AZNC’s ecology and landscape characteristics.

In response to the issues as stated above, this article selected the authoritative Globeland30 data set, and the classic and mature model for LULC simulation. Supported by the above data, the paper carries out quantitative analysis and spatial simulation of the ecological carrying capacity and landscape pattern in the study area. The paper expects to achieve the following two objectives:

(1) To construct a set of reasonable and feasible technical routes and parameter schemes for LULC simulations and quantitative measurement of BC.
(2) To analyze BC’s evolutionary patterns, trends, and crucial landscape indices in the AZNC.

## 2. Study Area, Data, and Methods

### 2.1. Study Area

**Figure 1:**
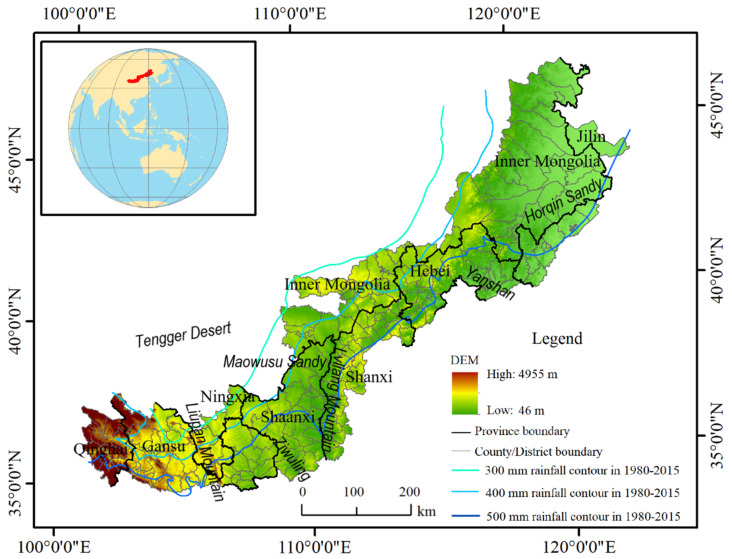
Location and topography of the study area.

The AZNC is situated in the region where eastern China’s semi-arid and arid agricultural areas meet western China’s semi-arid and arid pastoral areas (Figure 1). Its geographic range is 34°43′31″–46°57′46″ N, 100°57′11″–125°34′11″ E [38]. It roughly includes the northern Loess Plateau, the southern Mongolian Plateau, and the western part of the Northeast Plain. In terms of administrative division, it consists of 226 counties (banners, cities, and districts), 38 cities (leagues), and nine provinces (autonomous regions), namely Jilin, Liaoning, Inner Mongolia, Hebei, Shanxi, Shaanxi, Ningxia, Gansu, and Qinghai, with a total area of about 6.99 × 10^5^ km^2^.

The AZNC is situated in the temperate continental monsoon climate zone. The average annual precipitation ranges from 300 to 550 mm and steadily decreases from southeast to northwest. The interannual rainfall volatility is significant (CV = 27%). The study region’s topography gradually changes from plains to mountains and plateaus in a pattern that increases from northeast to southwest. The research area has a nested pattern of cultivated land and grassland. The percentage of cultivated land was 38.37%, while the grassland portion was 45.55% in 2020.

### 2.2. Source of Basic Data

The Globeland30 LULC data (2000, 2010, and 2020) were obtained from the China State Geospatial Information Center (http://www.globallandcover.com/ (accessed on 29 November 2022)). This dataset was created by Chen et al. [39] from the National Basic Geographic Information Center of China and is the world’s first 30 m resolution LULC product. The accuracy is over 80% and includes ten land cover categories. The Chinese government has donated the Globeland30 product to the UN, and all nations’ governments and scientists are welcome to use it. This paper created six land categories—cultivated land, forest, grassland, water, built up, and deserted land—by classifying the various land types in the research area (Table 1).

The key to simulating LULC change is to have accurate drivers and available datasets. Wang et al. [40] found critical influencing elements for LULC variations in the grassland area of northern China (Xilingol), including population, economy, livestock density, precipitation, temperature, and distance from urban and rural areas. A study by Xu et al. [41] in the agricultural region of northern China (northern Shanxi) showed that population density, per capita gross domestic product (GDP), precipitation, slope, and elevation were the main driving factors behind LULC variations. Taking into account the factors of ecology, production characteristics of the AZNC, and the history of ecological restoration project, we modified the previously chosen driving forces by adding the following three new indicators: gross agricultural output, gross output value of animal husbandry, and ecological construction investment. Finally, 13 LULC change driving factors and four categories were created (Table 2).

Specifically:

(1) In terms of physical geography, the SRTM DEM data of the study area was downloaded from the Geospatial Data Cloud website (http://www.gscloud.cn/ (accessed on 29 November 2022)) and further calculated to obtain the slope and slope direction.
(2) In terms of climate conditions, according to the global data set provided by CHIRPS Daily [42] and GLDAS-2.1 [43], this paper calculated the total annual rainfall and annual average temperature in the study area.
(3) In terms of accessibility, the road data in the study area was downloaded from the Geospatial Data Cloud website (http://www.gscloud.cn/ (accessed on 29 November 2022)) and the distribution of construction land in the study area was extracted from the GlobeLand30 dataset. Finally, the distance from any point in the study area to roads and building sites was calculated in ArcGIS.
(4) In terms of socio-economic development, the 1 km resolution population density and GDP data of the study area for 2010 and 2015 were downloaded from the China Resource and Environment Science and Data Center (http://www.resdc.cn/ (accessed on 29 November 2022)). Population density and GDP data for 2020 were obtained through a linear extrapolation method. We can get the year-end livestock inventory, gross agricultural output, and gross output value of animal husbandry from the statistical yearbooks of the provinces and cities in the study region [44,45,46,47,48,49,50,51] and divide them by the county area. Each province’s ecological engineering construction investment was obtained from the China Statistical Yearbook On Environmental [52]. Then use the area of each county as the weight to obtain each county’s ecological engineering construction investment. Eventually, the number of livestock per unit area, the gross agricultural output per unit area, the gross output value of animal husbandry per unit area, and the total investment in ecological construction based on the county were obtained by spatialization.

We processed the above data using nearest neighbor sampling and vector-to-raster conversion. Finally, the driving factor and LULC datasets in the study region are acquired with a resolution of 500 m.

### 2.3. Research Methodology 

#### 2.3.1. Technical Route

**Figure 2:**
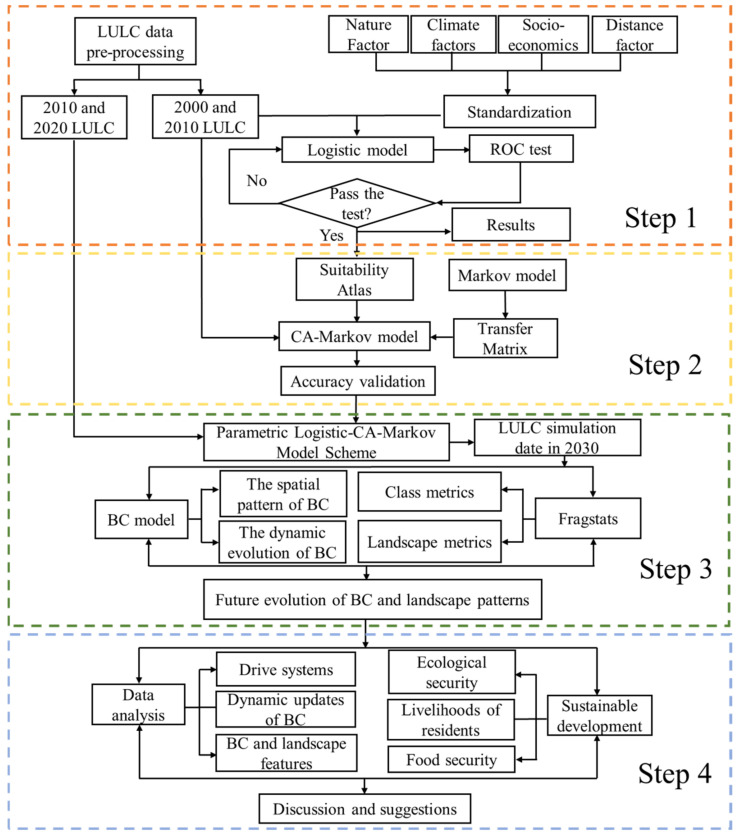
The overall technical route for this study.

The overall technical route includes 4 steps (Figure 2):

(1) The three GlobeLand30 periods of 2000/2010/2020 were cropped and reclassified under the size of the study region using ArcGIS, as well as spatialization and standardization factors that affect LULC changes, such as physical topography, climate condition, accessibility, and socio-economic development. After conducting a logistic regression analysis, the ROC curve was used to assess the model fitting effect.
(2) Based on the above logistic model, IDRISI32 [53] helped obtain a suitability atlas for the various theme elements. The CA-Markov module was used to simulate the 2010 LULC data to obtain the results of the LULC in 2020. In order to evaluate the accuracy and dependability of the Logistic-CA-Markov model, the simulated results were compared with GlobeLand30 2020 data.
(3) The CA-Markov module was applied to run simulations using the 2020 LULC data to get the 2030 LULC results based on the above Logistic-CA-Markov parameterization scheme. The spatial distribution of BC and its dynamic evolution process in the study area were examined using reliable institutions’ BC calculation parameter scheme. With the support of Fragstats software [54], a multi-scale landscape index calculation was carried out to analyze the dynamic evolution process of key landscape indices in the study area.
(4) Based on the above process, analysis and discussion were carried out. Suggestions were made from multiple dimensions, such as the driving index system of the simulation system, the dynamic update of BC accounting factors, the necessity for integrated analysis of BC and landscape characteristics, ensuring food security, improving farmers and herdsmen’s livelihood, and maintaining ecological security.

#### 2.3.2. Model Construction

By examining the neighborhood relationships of the CA model and putting the time–space synchronization simulation of LULC changes into practice, the traditional CA-Markov model improves spatial pattern simulation capabilities [55]. It is founded on the Markov model’s accurate prediction of the quantitative structure of the future LULC. The CA-Markov model, however, does not consider the potential effects of socio-economic and other factors associated with LULC, which reduces the predictability of the results [56]. This paper initially modified the conversion rules of the CA model by using the logistic regression analysis model to obtain the land suitability atlas. The three were combined with the Logistic-CA-Markov model, which was then applied for simulation.

(1) Logistic regression model

Logistic regression is a generalized linear regression analysis model used to estimate the likelihood of an event occurring. The regression equation is as follows:

(1) $$logit\left(p_{i}\right)=\ln\left(\frac{p_{i}}{1-p_{i}}\right)=\beta_{0}+\beta_{1}X_{1}+\beta_{2}X_{2}+\cdots +\beta_{n}X_{n}$$

where: $p_{i}$ is the probability of occurrence of land type *i*, $\beta_{0}$ is a constant, $\beta_{1},\beta_{2},\;\cdots ,\;\beta_{n}$ are regression coefficients, $X_{1},X_{2},\;\cdots ,\;X_{n}$ are driving factors, the value of $p_{i}$ ranges from 0 to 1, and the larger the value, the greater the probability of the occurrence of the land type.

(2) CA-Markov model

CA is a non-linear dynamical model that is usually discrete in time, space, and state [57]. CA consists of four basic elements: cell, cell space, cell neighbors, and cell rules, and cell transformation rules are its core part. Applying its transformation rules, the spatio-temporal processes of complex systems can be modelled based on temporal causality and spatial interactions [58]. The model described is as follows:

(2) $$S_{\left(t+1\right)}=F\left(S_{\left(t\right)},N\right)$$

where: S is the set of cell states, *t* and *t* + 1 are different times, *F* is the transformation rule of cell states, and *N* is the neighborhood of the cell. 

The Markov model is a classical discrete-time stochastic model used for LULC change simulation [59]. Its main principle is to predict the future LULC distribution by calculating the area and probability of transfer between classes based on the past and current LULC distribution. Its process of modelling LULC change is as follows:

(3) $$S_{\left(t+1\right)}=P\cdot S_{\left(t\right)}$$

(4) $$P_{ij}=\left[\begin{array}{cccc} p_{11} & p_{12} & \cdots & p_{1n}\\ p_{21} & p_{22} & \cdots & p_{2n}\\ \vdots & \vdots & \vdots & \vdots \\ p_{n1} & p_{n2} & \cdots & p_{nn} \end{array}\right]$$

where: $p_{ij}$ is the state transition probability matrix, which represents the probability of transforming class *i* land into class *j* land, $0\leq p_{ij}<1$ and $\sum_{j=1}^{n}p_{ij}=1,\left(i,j=1,\;2,\;3,\ldots ,n\right)$.

(3) BC model

BC is the maximum limit of the existence of a certain number of individuals under a specific environmental condition and its measurement unit is gha or ghm^2^. The BC of a region is a measure of the ecological assets available in the region (including farmland, forest, pastures, fisheries, and built up land) and their capacity to produce renewable resources and ecological services [60]. The BC calculation model is as follows:

(5) $$BC=\overset{n}{\underset{i=1}{\sum }}A_{i}\times YF_{i}\times EQF_{i}=\overset{n}{\underset{i=1}{\sum }}A_{i}\times BF_{i}$$

where: n is the number of types of LULCs in the area, $A_{i}$ is the area of land of type *i* biological productivity (in ha), $YF_{i}$ is the Yield Factor of land of type *i* biological productivity (in 1), $EQF_{i}$ is the Equivalence Factor of land of type *i* biological productivity (in gha/ha), $BF_{i}$ is the BC per unit area of land of type *i* production (in gha/ha).

This study set the YF of built-up and deserted land with extremely low natural productivity as zero according to the actual conditions of the study area. YF and EQF are quoted from the Global Footprint Network (GFN) and World Wildlife Fund (WWF) [61,62], and missing data are replaced with data from adjacent years. The corresponding indicators in 2030 are assumed to be the same as in 2020, and the specific YF and EQF values can be found in Table 3.

(4) Landscape Index

The landscape index is one of the most popular techniques for analyzing landscape patterns. It dramatically reduces the amount of information in landscape spatial patterns, and quantitatively expresses the characteristics of the structure, dynamic changes, and spatial configuration [63]. The three scales of the landscape index are patch, class, and landscape metrics. With the support of Fragstats software, we performed index calculations on two scales, the landscape metrics and the class metrics (Table 4).

#### 2.3.3. Accuracy Verification

In order to assess whether the logistic regression model is competent for the preparation of suitability atlases, this paper used the Relative Operating Characteristic (ROC) curve proposed by Pontius et al. [64] to evaluate the fitting effect. It is generally accepted that when the area value of the ROC curve is greater than 0.7, the model can be judged as good enough to meet the needs of the study [11]. This study used the Kappa coefficient as an indicator for the consistency test of the spatial simulation of the Logistic-CA-Markov model. The formula is as follows:

(6) $$Kappa=\frac{P_{0}-P_{c}}{1-P_{c}}$$

where: *P_0_* is the proportion of predicted consistent raster to the actual total raster and *Pc* is the proportion of simulated consistent predicted raster in the random state. When 0 < *Kappa* < 0.4, the model simulation result is considered to be poor; when 0.4 ≤ *Kappa* < 0.75, the simulation prediction accuracy is considered to be general; when *Kappa* ≥ 0.75, the model simulation effect is considered to be very good, and the accuracy test is passed [65].

## 3. Results

### 3.1. Model Verification and Accuracy Evaluation

The logistic model can obtain the logistic regression equation between each driving factor and land type. The following table displays the coefficient (β) of each factor in the equation as well as the ROC of the equation (Table 5). All land types have ROC values above 0.90, which are significantly higher than 0.75. This demonstrated the validity and utility of the driving factors used in this study. These drivers were used to build a logistic regression model with strong explanatory power for the variation in LULC.

The nature and absolute value of the regression coefficient β can be used to judge whether the driving factor has a driving effect on a specific land change and the magnitude of the driving force. When the absolute value of the regression coefficient is greater than 0.5, we consider this factor as the main influencing factor.

The above table makes clear that there is very little correlation between population density and GDP and different types of land changes in the area. The absolute values of these two factors’ regression coefficients are usually less than 0.005 or 0.001. However, the LULC changes are greatly influenced by physical geography (slope), climate conditions (precipitation, temperature), and socio-economic development (distance to built-up land, distance to county road, gross agricultural output, etc.). Regression coefficients for these factors usually have absolute values exceeding 2.0.

Using the previous logistic regression model, this study configured the CA-Markov simulation parameters (5 × 5 filters, 10 cycles). Based on the Globeland30 data in 2000 and 2010, this paper simulated the spatial distribution of LULC in 2020 and compared it with the real GlobeLand30 2020 data. The findings revealed that, in contrast to the actual GlobeLand30 2020 data, the simulated Kappa coefficient is 0.805. Additionally, the simulated results are very similar to the spatial distribution pattern of the original GlobeLand30 2020 data, implying that the CA-Markov and logistic models perform better. Thus, driving factors and specific parameters scheme adopted in this study are reliable and can be used for LULC simulations.

### 3.2. Spatial Distribution and Dynamics of LULC

**Figure 3:**
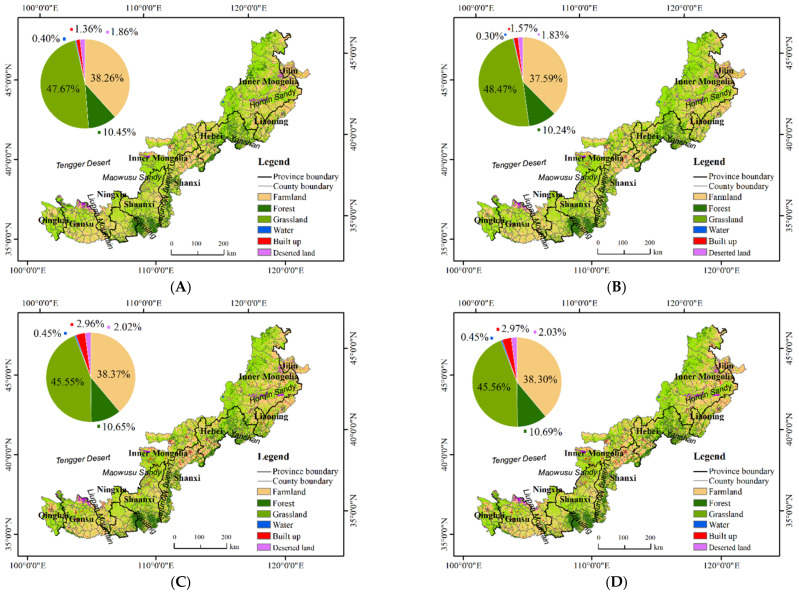
Spatial distribution of land use in the Agro-Pastoral Zone of Northern China from 2000 to 2030. (**A**) 2000; (**B**) 2010; (**C**) 2020; (**D**) 2030.

The LULC simulation results for the study area in 2030 (Figure 3D) show that the spatial distribution pattern of LULC in 2030 does not change significantly compared to that in 2000–2020 (Figure 3A–C). The study area is dominated by cultivated land (26.8 × 10^4^ km^2^, 38.30%), grassland (31.8 × 10^4^ km^2^, 45.56%), and forest (7.5 × 10^4^ km^2^, 10.69%). The areas of built-up land, deserted land, and water are very small (2.1 × 10^4^ km^2^, 2.96%; 1.4 × 10^4^ km^2^, 2.03%; 0.3 × 10^4^ km^2^, 0.45%, respectively). Cultivated land and grassland are widely distributed and are the dominated land use types. Forests are relatively densely distributed in mountainous areas such as Yanshan in northern Hebei, Lvliang Mountain in northern Shanxi, and Ziwuling in central Shaanxi. The deserted land is mainly distributed in the Horqin Sandy Land in Inner Mongolia, the Maowusu Sandy in Shaanxi and the edge of the Tengger Desert. Water is sporadically distributed throughout the study area.

### 3.3. Spatial Distribution and Dynamics of BC

**Figure 4:**
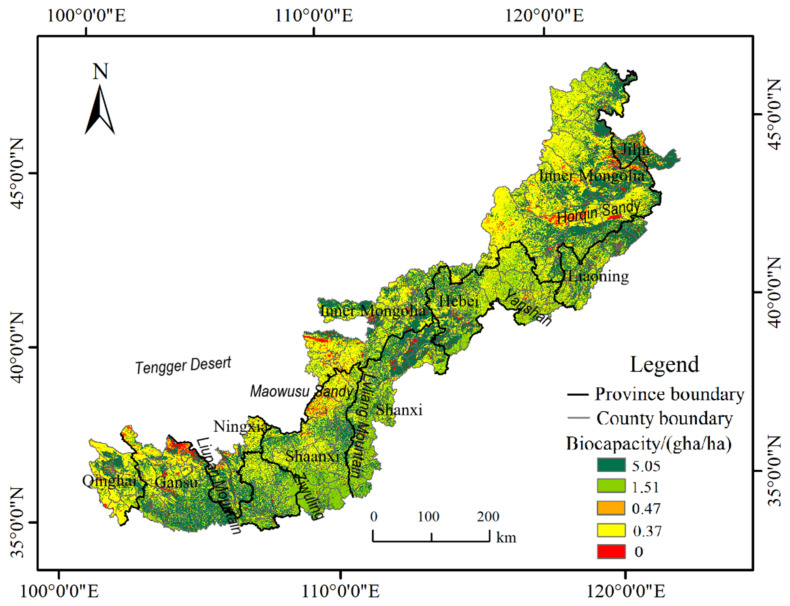
Spatial distribution of BC in the Agro-Pastoral Zone of Northern China in 2030.

In 2030, the spatial distribution of BC in the study area shows a high coincidence with that of LULC (Figure 4). The areas with high BC (dark green) are mainly distributed in the cultivated land. The areas with a medium value of BC (light green) correspond well to the spatial distribution of forest. The low BC area (orange, yellow) mainly has a good spatial correspondence with the grassland. The BC with zero value area (red) corresponds to the Horqin Sandy, the Maowusu Sandy, the edge of the Tengger Desert, and cities that are scattered.

**Figure 5:**
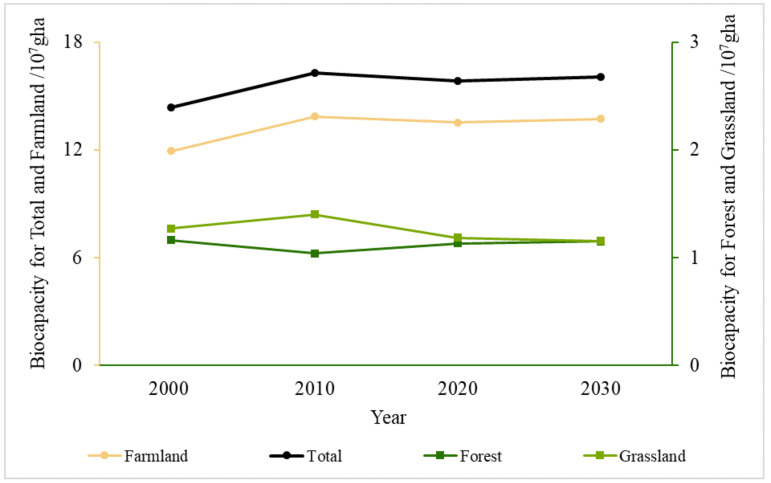
Change tendency of BC in the Agro-Pastoral Zone of Northern China from 2000 to 2030.

Temporally, the BC of the AZNC showed an upward tendency from 2000 to 2030 (Figure 5). The study area’s multi-year average BC is 1.57 × 10^8^ gha, and the annual growth rate is +4.55 × 10^6^ gha/a. The BC rises severely between 2000 and 2010 (+1.93 × 10^7^ gha, +13%) and decreases slightly from 2010–2020 (−0.46 × 10^7^, −3%). From 2020 to 2030, BC is expected to exhibit a moderate rising trend. The BC of cultivated land exhibits a more pronounced increasing trend overall (+5.05 × 10^6^ gha/a). The BC of grassland exhibits a slight declining trend (+0.48 × 10^5^ gha/a), whereas the BC of forest exhibits a slight increasing trend (−5.6 × 10^5^ gha/a). The BC of the water is small and stable at about 1.36 × 10^5^ gha.

### 3.4. Dynamic Changes in the Landscape Pattern

From 2000 to 2030, the study area exhibits a trend toward fragmentation, miniaturization, and homogenization of landscape patches, a decrease in the degree of landscape aggregation, and an increase in diversity. 

**Figure 6:**
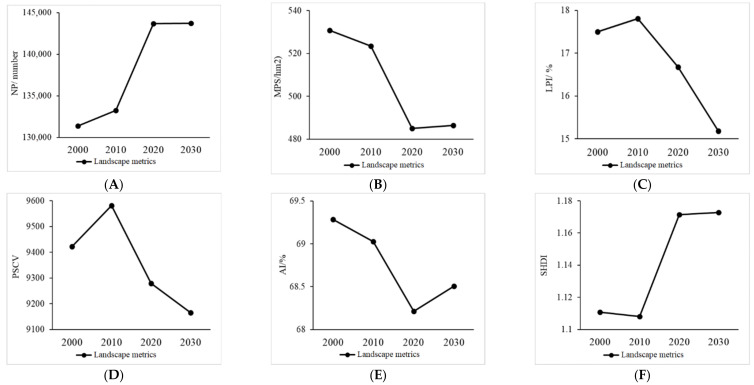
Changes in landscape metrics from 2000 to 2030. (**A**) NP; (**B**) MPS; (**C**) LPI; (**D**) PSCV; (**E**) AI; (**F**) SHDI.

From the perspective of landscape metrics, during 2000–2030, the NP in the research area considerably grew (+9.37%), whereas the MPS significantly declined (−8.62%) (Figure 6A,B). This shows a trend of landscape fragmentation and miniaturization in the study area. A significant decrease follows a slight increase in the LPI and PSCV (Figure 6C,D), indicating a miniaturization and homogenization trend of the landscape. At the same time, the AI exhibits an overall declining tendency with an initial sharp decrease and a subsequent minor gain (Figure 6E). The SHDI has an ascending tendency with an initial very modest fall followed by a considerable gain (Figure 6F). The results reflect the tendency of fragmentation at the landscape metrics.

**Figure 7:**
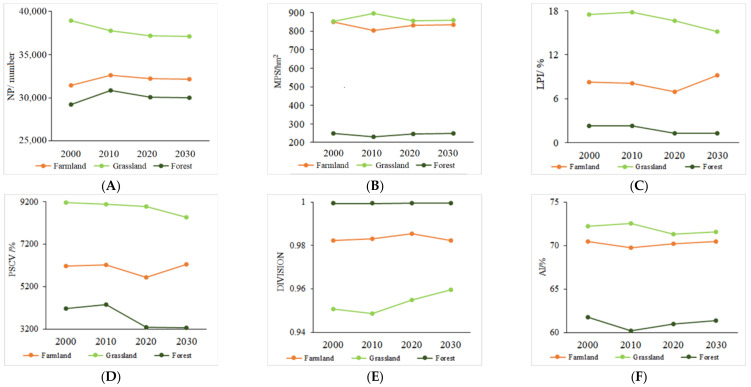
Changes in class metrics from 2000 to 2030. (**A**) NP; (**B**) MPS; (**C**) LPI; (**D**) PSCV; (**E**) DIVISION; (**F**) AI.

From the perspective of class metrics (Figure 7), the changes of the three main land types in the study area differ from the landscape metrics changes. The most distinctive feature is that, while the NP of grassland decreases from 2000 to 2030, the NP of cultivated land and forest increases (Figure 7A). The MPS of the three land types does not change much in different periods, but the MPS of grassland and cultivated land is significantly higher than that of forest (829.0 hm^2^, 865.0 hm^2^, and 244.2 hm^2^, respectively) (Figure 7B,C). The obvious decline of MPS at the regional scale is mainly caused by landscape fragmentation. The PSCV of grassland and forest showed a significant downward trend, indicating that the grassland and forest showed a trend of the uniform patch area (Figure 7D). The cultivated land showed a significantly low value in 2020, which is similar to the values in other years. The DIVISION for all three main land types shows a slight increase, while the AI shows a slight decrease (Figure 7E,F).

## 4. Discussion

### 4.1. Uncertainty of the Study

This study focuses on the driving mechanisms of LULC change based on the logistic model. The results demonstrated that population density and GDP have a slight driving relationship with the change of each type of land in the AZNC. In contrast, the variance of LULC is significantly influenced by physical geography (slope), climate conditions (precipitation, temperature), and socio-economic development (distance to built-up land, distance to county road, gross agricultural output, etc.). The results are highly consistent with research by Li et al. [66], which was conducted in the Gansu province of China—an arid and semi-arid area, and concluded that physical and geographical factors such as temperature, precipitation, and slope are the main driving factors of LULC changes. However, the research of Wang [40] and Xu et al. [41] indicated that population density and economic development have a greater impact on the changes in LULC in typical grassland livestock and dry farming areas, which are different from our study. The reason may be that other studies generally carry out simulation analysis on smaller spatial scales (such as county scale or city scale, with an area usually smaller than 2 × 10^5^ km^2^), where population density and per capita GDP will have larger gradient changes on finer grids. In our study, the analysis is carried out on a larger spatial scale (climate zone, physical geographic zone, with an area of about 7 × 10^5^ km^2^), where the level of economic and social development in different regions is not very different due to low population density. The spatial gradients of the above two indicators are not obvious in coarse-resolution raster data. This may be the reason that these two factors are not sensitive to the effect of regional LULC changes.

At the same time, unique industrial structure, large-scale investment in ecological protection, and ecological construction projects have important influences on the study of the agro-pastoral cross belt and similar areas. Based on the comparison of two CA-Markov models, we found that the addition of driving factors such as the output value of agricultural and animal husbandry, livestock density, and investment in ecological construction has an important impact on the accuracy of logistic regression analysis (Table 5). After adding the above three factors, the ROC of each category increased. This shows that in the AZNC, in addition to factors such as physical geography and traffic accessibility, in comparison to industrial and commercial developed regions, the study area’s special industrial structure and land development investment structure factors, namely: farming output, animal husbandry output, and investment in ecological construction, have a more important impact on the changes in regional LULC. This is an important factor for conducting LULC simulations in regions with similar natural and socio-economic conditions.

In addition to the land type area factor, the YF and the EQF also are key parameters determining BC. The results of different studies varied slightly since YF and EQF involve collecting, correcting, and timely updating agricultural production and prices in different countries and regions around the world [67]. In this study, we used YF and EQF data provided by GFN and WWF. It would require a more thorough empirical study to determine whether the parameters offered by these foreign institutions are appropriate for the actual situation across China. Liu et al. [26,27] offers helpful examples. However, sadly the research was only done up until 2001. This study assumed that the YF and EQF in 2030 will be the same as in 2020 this research. Although necessary, this assumption will indeed differ from the actual outcome. We believe that our current projection of BC in 2030 is likely to be underestimated (by 7–10%), under the assumption that YF and EQF can be extrapolated from the time series trend between 2000 and 2020.

### 4.2. Impact and Recommendations

Many studies have shown that a region’s quality of ecosystem services is closely related to ecosystems’ typological composition and spatial configuration characteristics. Haddad et al. [68] studied the impact of habitat fragmentation on global forest cover. The results show that habitat fragmentation reduces biodiversity by 13–75%, degrading ecosystem functions by reducing biomass and altering nutrient cycling. By taking the Shenzhen city of China as an example, Li et al. [69] studied the impact of landscape patterns on the urban heat island effect. The study results show that landscape composition significantly impacts the urban heat island effect more than landscape configuration. Moreover, big cities’ urban heat land effect can be alleviated by standardizing landscape composition. From 2000 to 2030, it benefited from a series of high-intensity ecological protection and ecological restoration projects having been planned by the Chinese government since the late 1990s. At the same time, the food security, and social and economic development of the study area was considered. The changes in LULC in the AZNC are mainly manifested as follows: a significant expansion of construction land (+118.38%), an increase in the area of forest land (+2.34%) and cultivated land (+0.09%), and a decrease in the area of grassland (−4.44%). Affected by the change in LULC, although the BC of the AZNC has increased, the ecological landscape has clearly shown a trend of miniaturization, fragmentation, and dispersion. Currently, this work only systematically evaluates changes in the ecological landscape index and the BC index. However, it does not realize an integrated analysis of the ecological landscape characteristic index, ecosystem carrying capacity, and ecosystem service and function. Therefore, in future research, constructing a comprehensive index system that reflects both landscape change and BC change should be considered. At the same time, the coupling and decoupling analysis of BC or ecosystem service indices, and ecological landscape indices at different spatial scales should be carried out. Moreover, how to take into account the overall pattern of ecological landscape while enhancing the regional ecological carrying capacity is also an essential topic for the future development of the study area.

In addition, cultivated land is a prerequisite for national food security since it serves as the primary material base for food production. The quick and dramatic decline of cultivated land would worsen issues with food security and put farmers’ and herders’ livelihoods in peril. The AZNC must consider ecological advantages, food security, and the livelihoods of farmers and herders because it is an area with a medium population density, complicated land use patterns, and exceptional ecological services. It is vital to produce distinctive agricultural goods, economic forests, orchards, and high-quality animal husbandry and allocate resources across cultivated land, grassland, and forest as efficiently as possible. How to fully exploit the agro-pastoral zone’s geographic advantages while stabilizing and improving the regional BC must be considered, to promote ecological farming and animal husbandry, reduce regional poverty, prevent a return to it, and get the most out of the environment. This is a crucial practical responsibility for local land development businesses and institutions as well as a crucial study topic for regional land planning scholars. 

## 5. Conclusions

This paper applies Logistic and CA-Markov models to simulate the spatial distribution of LULC in the AZNC in 2030, based on the global common LULC dataset and authorized government socio-economic statistics. The evolution patterns of BC and landscape patterns in the region from 2000–2030 are analyzed. The study is an extension and deepening of the classic LULC changes study.

Our research shows that population density and GDP have a weak impact on the LULC change in the region. In contrast, factors such as output value of agricultural and animal husbandry sectors, livestock density, and ecological construction investment significantly affect the LULC change in the region. From 2000 to 2030, the regional BC showed an upward trend due to the policy of returning farmland to forest and grassland. However, the ecological landscape showed a trend of miniaturization and fragmentation. 

Our study demonstrates the reliability of the Logistic-CA-Markov model and its driver and parameterization scheme for LULC simulations in the AZNC. At the same time, we also point out the problems that may result from the uncertainties in the YF and EQF factors during BC calculations. This study highlights the necessity of a regionally integrated analytical framework for BC and natural landscape patterns. Furthermore, it emphasizes the importance of scientific and reasonable land development planning and practice. In addition, we put forward reasonable suggestions for balancing food security, farmers’ and herders’ livelihoods, and regional ecology, which is a crucial issue for sustainable development of ANZC in the future.

## Figures and Tables

**Table 1:** LULC classification system in study area.

Code	LULC Type	LC ID	Globeland30 Classification System
1	Cultivated land	10	Cultivated land
2	Forest	20	Forest
3	Grassland	30	Grassland
		40	Shrub land
		50	Wetland
4	Water	60	Water bodies
5	Built up	80	Artificial surfaces
6	Deserted land	90	Bare land
		100	Permanent snow and ice

**Table 2:** Driving factors of LULC change in AZNC.

Type	Variables	Scale/Resolution	Year	Data Sources
Physical Geography	1. Elevation	90 m	2008	http://www.gscloud.cn/ (accessed on 29 November 2022)
	2. Aspect	90 m	2008	http://www.gscloud.cn/ (accessed on 29 November 2022)
	3. Slope	90 m	2008	http://www.gscloud.cn/ (accessed on 29 November 2022)
Climate Conditions	4. Total precipitation	0.05 arc degrees	2010/2020	https://developers.google.com/earth-engine/datasets (accessed on 29 November 2022)
	5. Average annual temperature	0.25 arc degrees	2010/2020	https://developers.google.com/earth-engine/datasets (accessed on 29 November 2022)
Accessibility	6. Distance to built-up land	30 m	2010/2020	http://www.globallandcover.com/ (accessed on 29 November 2022)
	7. Distance to county road	500 m	2015	http://www.gscloud.cn/ (accessed on 29 November 2022)
Socio-Economic Development	8. Population density	1000 m	2010/2020	http://www.resdc.cn/ (accessed on 29 November 2022)
	9. GDP	1000 m	2010/2020	http://www.resdc.cn/ (accessed on 29 November 2022)
	10. Livestock density	County	2010/2020	Produced by the authors
	11. Gross agricultural output	County	2010/2020	Produced by the authors
	12. Gross output value of animal husbandry	County	2010/2020	Produced by the authors
	13. Ecological construction investment	County	2010/2020	Produced by the authors

**Table 3:** Values of YF and EQF in different years in the study area.

LULC Type	2000		2010		2020		2030	
	YF	EQF	YF	EQF	YF	EQF	YF	EQF
Cultivated land	2.12	2.11	2.21	2.39	2.02	2.50	2.02	2.50
Forest	1.18	1.35	1.18	1.24	1.18	1.28	1.18	1.28
Grassland	0.81	0.47	0.81	0.51	0.81	0.46	0.81	0.46
Water	1.27	0.35	1.27	0.41	1.27	0.37	1.27	0.37

**Table 4:** Indices and their meaning for landscape pattern.

Landscape Index	Scale	Acronyms	Unit	Description
Number of Patches	C/L	NP	Number	NP reflects the number of patches of landscape type, with a large NP indicating a high degree of landscape fragmentation.
Mean Patch Size	C/L	MPS	hm^2^	MPS is the average area of all patches or a particular patch in a landscape and can be used to measure the degree of fragmentation of the landscape.
Largest Patch Index	C/L	LPI	%	LPI is the proportion of the entire landscape area occupied by the largest patch of each patch type, and LPI can be used as a measure of the dominant landscape type.
Landscape Division Index	C/L	DIVISION	-	DIVISION reflects the extent to which patches of the same category are segmented and isolated from each other, and takes on a range of values: 0 ≤ COHESION <1.
Patch Size Coefficient of Variation	C/L	PSCV	-	PSCV is used to characterize the degree of dispersion of the patch area.
Aggregation Index	C/L	AI	%	AI is used to describe the degree of agglomeration of different landscape elements, and takes the following values: 0 ≤ AI < 100.
Shannon’s Diversity Index	L	SHDI	-	SHDI reflects the degree of homogeneity and complexity of the distribution of the different landscape types in the region and takes the value range: SHDI ≥ 0.

Note: C is the class metrics and L is the landscape metrics.

**Table 5:** Logistic regression model for each land type.

Type	Variables	Cultivated Land	Forest	Grassland	Water	Built Up	Deserted Land
Physical Geography	1. Elevation	−0.094	0.707	0.481	1.635	4.503	1.343
	2. Aspect	0.46	−0.31	−0.156	1.069	0.671	−0.267
	3. Slope	−1.905	4.649	2.077	1.113	5.589	−3.064
Climate Conditions	4. Total precipitation	1.007	4.828	−2.209	−5.625	−0.597	−8.157
	5. Average annual temperature	2.007	−1.035	−1.299	6.867	3.768	2.721
Accessibility	6. Distance to built-up land	−1.314	1.188	1.918	3.025	−21.389	2.106
	7. Distance to county road	−0.158	1.684	2.29	0	7.457	5.319
Socio-Economic Development	8. Population density	−0.001	0.001	0.001	−0.001	0.005	−0.002
	9. GDP	0.002	−0.001	−0.002	−0.001	−0.004	0.001
	10. Livestock density	0.317	−8.978	0.683	−1.522	0.774	0.305
	11. Gross agricultural output	−4.112	1.637	2.218	−1.932	−0.081	2.13
	12. Gross output value of animal husbandry	5.105	6.993	−3.428	0.662	1.3	−2.01
	13. Ecological construction investment	−1.31	0.348	0.527	−2.012	−1.993	−0.425
	ROC (regard to all index)	0.939	0.962	0.944	0.927	0.969	0.955
	ROC (without regard to index 11, 12, 13)	0.924	0.959	0.936	0.921	0.958	0.955

Note: The darker the color, the larger the regression coefficient. Red means negative correlation, blue means positive correlation.

## Data Availability

Not applicable.
